# The principle and physical models of novel jetting dispenser with giant magnetostrictive and a magnifier

**DOI:** 10.1038/srep18294

**Published:** 2015-12-16

**Authors:** C. Zhou, J.H. Li, J.A. Duan, G.L. Deng

**Affiliations:** 1 School of Mechanical and Electrical Engineering and State Key Laboratory of High Performance Complex Manufacturing, Central South University, Changsha 410083, China

## Abstract

In order to develop jetting technologies of glue in LED and microelectronics packaging, giant-magnetostrictive-material (GMM) is firstly applied to increase jetting response, and a new magnifying device including a lever and a flexible hinge is designed to improve jetting characteristics. Physical models of the jetting system are derived from the magnifying structure and working principle, which involves circuit model, electro-magneto-displacement model, dynamic model and fluid-solid coupling model. The system model is established by combining mathematical models with Matlab-Simulink. The effectiveness of the GMM-based dispenser is confirmed by simulation and experiments. The jetting frequency significantly increases to 250 Hz, and dynamic behaviors jetting needle are evaluated that the velocity and displacement of the jetting needle reaches to 320 mm•s-1 and 0.11 mm respectively. With the increasing of the filling pressure or the amplitude of the current, the dot size will become larger. The dot size and working frequency can be easily adjusted.

In recent years, dispensing technology, including jetting and contact-based dispensing, has been widely used in many industrial applications, such as an assembly of micro-electronics, optoelectronics and LEDs^1^,^2^,^3^,^4^,^5^. Contact-based dispensing technique can be classified into three types: time-pressure, rotary-screw and positive displacement^6^. In contact-based dispensing method, repeatability requires the same dispensing gap (the gap between the needle and the substrate or PCB) of each dot. Maintaining such a constant dispensing gap needs a positioning system that can move the nozzle up and down accurately during the dispensing process. Thus, the cycle time increases and the process become complicated^7^,^8^,^9^,^10^,^11^. To solve the problem, some experts studied a pneumatic jetting dispensing technique, which is used widely now. However, pneumatic jetting dispensers depend on electromagnetic valves of high frequency. The electromagnetic valves have only a lifespan of few months and the price is high. For these reasons, some experts engaged in PZT (Pb(Zr_x_Ti_1-x_)O_3_—a kind of piezoelectric ceramic) driving dispenser research, and made great achievements^12^,^13^. Compared with PZT, GMM has larger energy density and therefore, can supply a larger driving force. GMM is mainly used for sound recognition and micro-displacement driving nowadays^14^,^15^.

Therefore, this work was undertaken to examine a new type of dispenser based on GMM drive, and design the magnifying device to improve the dynamic behaviors. Furthermore, complex models and superior performance of the new system are discussed.

## Methods

### Innovative configuration & principle of the GMM-based jetting dispenser

**Figure 1:**
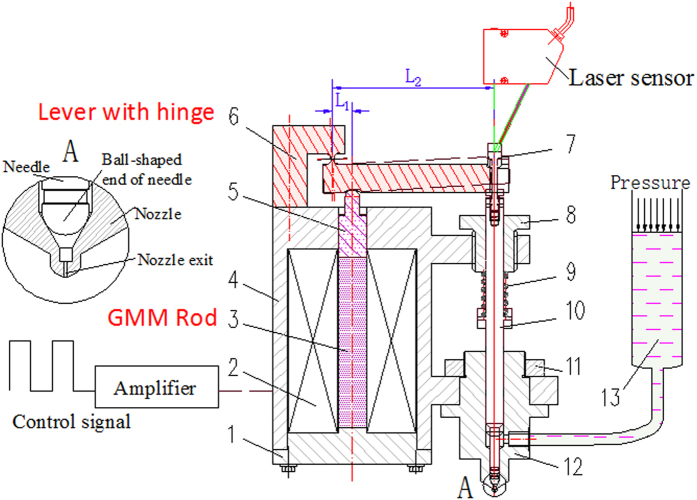
New structure of the GMM-based jetting dispenser. 1-End cap, 2-Coil, 3-GMM Rod, 4-Shell, 5-Transmission bar, 6-Lever with hinge, 7-Adjusting nut, 8-Spring preload adjusting block, 9-Spring, 10-Needle, 11-Nut, 12-Nozzle, and 13-Syringe.

Magnetostriction is a property of ferromagnetic materials that causes them to change their shape or dimensions during the process of magnetization^16^. The extension ratio of GMM is very small. As the displacement of the needle driven by the GMM rod directly is not large enough, the glue cannot be jet. In this experiment, a lever with flexible hinge is used for magnifying displacement and the alternative magnetic field was stimulated by an electromagnetic coil. As shown in Fig. 1, part 6 is the lever with a flexible hinge, which is made of manganese steel. One end of the lever is fixed to the shell (part 4), and the other end can rotate around the flexible hinge. The lever is elaborately designed to transmit force and magnify displacement.

The designed dispenser includes a magnetostrictive actuator and a glue injector. The magnetostrictive actuator mainly contains a shell (part 4), a coil (part 2), a GMM rod (part 3), an end cap (part 1), a lever with hinge (part 6), an adjusting nut (part 7), a spring preload adjusting block (part 8), a spring (part 9), a needle (part10) and a transmission bar (part5). The glue injector contains a nozzle (part 12), a syringe (part 13) and a needle (part10).

A spring (part 9) and the flexible hinge (part 6) are used to make mechanical bias. The stiffness of the spring is 24 N/mm. The force is transmitted through the needle, the nut (part 7), the lever and the transmission bar. The nut is used to adjust mechanical bias force. The spring can also make the needle compacting on the nozzle in order to prevent glue leaking out, when the dispenser does not work.

Bias magnetic field is always used in magnetostrictive actuator for precision displacement, as it would affect the relative elongation of GMM. In this experiment, the accuracy of displacement is not so important^17^,^18^,^19^. By considering the complexity, volume and the mass, the first generation dispenser is designed without the coil used to make bias magnetic field.

Alternative magnetic field stimulated by the coil (part 2) will make GMM rod extend or shorten. The adjusting block (part 8) is used to adjust the spring preload. Pressure in syringe makes glue filled continuously. Besides the geometry parameters of the dispenser, the voltage across the coil, the duty circle of the controlling signal, pre-pressure of the spring, pre-pressure of the GMM rod, pressure of glue supplying in syringe are adjustable parameters. If these parameters match, the dispenser can work steadily, otherwise, the glue would accumulate near the nozzle exit or the dispenser cannot work.

The system’s workflow is as follows: When the current is on, the coil becomes magnetic and the GMM rod in the coil extends. The block on the GMM rod moves up with the rod. Then, the lever rotates around the center of the hinge. The needle moves upward with the lever and the spring is compressed at the same time. The needle stays at the highest point for 3 to 10 micro-second. Hence, there is sufficient time for the glue to be filled into the chamber of the nozzle. After that, the magnetic field of the coil disappears as the current is turned off, and the GMM rod returns to the initial state. Elastic potential energy from the spring is converted into kinetic energy of needle immediately. The needle moves downward rapidly. Therefore, shearing between the needle and glue makes the glue in the chamber get thin and flow. When the needle strikes on the nozzle, a glue dot is jetted out from the nozzle and the whole cycle starts again.

**Figure 2:**
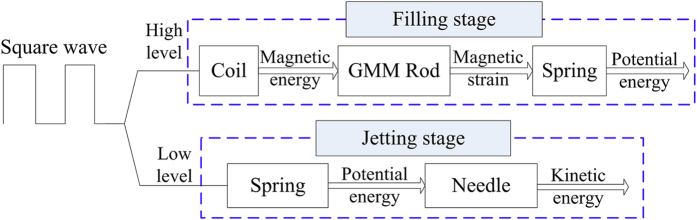
Energy transformation chart.

According to the working principle, the working process can be divided into filling stage, jetting stage and an interval. When the needle begins moving upward, the filling stage starts. When the needle begins moving downward, the jetting stage starts and the filling stage ends at the same time. After the jetting stage, an interval comes. Energy transmission during the whole process is shown in Fig. 2. The square wave is used to activate the coil. The frequency, amplitude and duty circle of the square wave can be adjusted to match other parameters of the dispenser.

**Figure 3:**
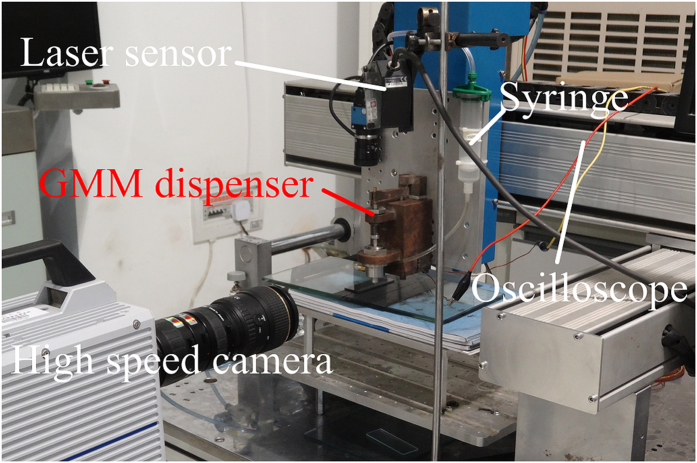
Experimental bed.

In order to evaluate the dispensing performance, an observation system and a measurement system are set up as shown in Fig. 3. Kyence laser sensor (LK-G80) is used to measure displacement of the needle. The sampling frequency of the sensor can achieve 50 kHz, and the accuracy can achieve 0.2 μm. By the sensor, instantaneous displacement can be recorded. The velocity can be obtained through solving the displacement difference. When dispensing experiment is carried out, a high speed camera (Brand: Photron, Model: FastCAM Sa1.1) is used to record the dispensing process.

### Physical models of new jetting dispenser

The new GMM-based jetting dispenser integrates electromechanical and hydraulic sub-systems. Electric energy, magnetic energy, potential energy and kinetic energy are transformed and transmitted through these sub-systems. All energy variation of the dispenser system is determined by corresponding laws. According to corresponding laws, mathematic models are established. Then, with these models, a numerical simulation is presented.

### Circuit model

Power is transformed into excitation current with circuit system of dispenser. The delayed time of the control circuit (within 100 nanosecond) is neglected when analyzed, for it is very short. Effect of temperature variation is also ignored. According to Kirchhoff’s voltage law, the equation of the circuit system is as follow,


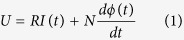


Where *U* is the voltage of the coil, *I* is the current of the coil, *N* is the turn number of the coil, *ϕ(t)* is the magnetic flux, *R* is the total resistance of the circuit. When starting, *R* is the resistance of the coil. When freewheeling, *R* is the summation of resistance of the coil and freewheeling resistance.

### Electro-magneto-displacement model

The coil can transform current into magnetic field. The power of coil was stimulated by an amplifier (LVC5050 amplifier), which was controlled by inputting signal.

Magneto motive force (MMF) was defined in electromagnetics as,


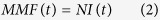


Where *N* is the turn number of coil, and *I(t)* is current.

Taking effects of eddy current into consideration, MMF is as follows,


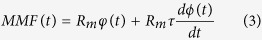


Where *R*_*m*_ is reluctance of GMM rod, *τ* is the eddy current time constant (*τ* = 0.0005 s), and *ϕ*(*t*) is the magnetic flux.


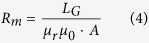



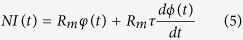


Where *L*_*G*_ is the length of GMM rod. *A* is sectional area of GMM rod. The bearing capacity will be enhanced with the increase of sectional area. *μ*_*r*_ is relative permeability. *μ*_*0*_ is permeability of vacuum.

*H*(*t*) is magnetic intensity, which can be calculated as follows,


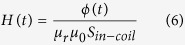


Where *S*_*in_−_coil*_ is the inner cylindrical surface area of the coil.

GMM strain and magnetic intensity table is shown in table 1, when the pre-stress is 6.0MPa (The data is provided by Taizhou Jiaoguang rare earth material Co., Ltd). A corresponding stain can be obtained for a certain magnetic intensity from the table.

The force and the elongation of the GMM rod are coupled. The force goes down when the elongation increases. For quasi-static load, GMM rod can be simplified as elastomer^20^. The force of GMM rod is calculated by the following formulas,


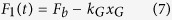



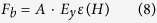


Where *k*_*G*_ is the stiffness of GMM rod, *x*_*G*_ is elongation indicator of GMM rod, *F*_*b*_ is GMM rod outputting force, when the displacement is zero, *ε*(*H*) is magnetic strain rate of GMM. *E*_*y*_ is young modulus of GMM rod.

### Dynamic model of the dispenser

**Figure 4:**
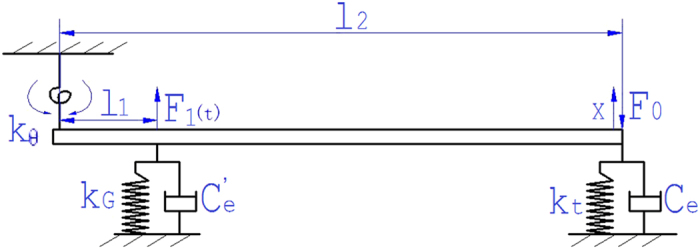
Force diagram.

The forced diagram of GMM rod, lever, needle and spring is shown in Fig. 4, and the dynamic of the system can be expressed in formula (9).


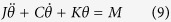


Where *J* is the moment inertia of the system, *C* is equivalent damping, *K* is equivalent stiffness coefficient of the system, *M* is torque suffered by the lever, and *θ* is angular displacement.

*J*, *K*, and *M* can be calculated as follows,


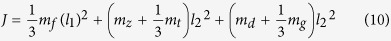



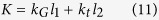



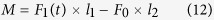


In the above, *m*_*f*_ is the mass of the lever, *m*_*f*_ = 50.6 g, *m*_*z*_ is the mass of the needle, *m*_*z*_ = 17.6 g, *m*_*t*_ is the mass of the spring, *m*_*t*_ = 3.32 g, *m*_*g*_ is the mass of the GMM rod, *m*_*g*_ = 43.4 g, *m*_*d*_ is the mass of the tapered block, *m*_*d*_ = 10.6 g, *k*_*t*_ is the stiffness of the spring, *k*_*t*_ = 20.6 N•mm^−1^.

Damping of the system includes two parts: The first is the viscous damping of glue to the needle; the other part is damping among the GMM rod, magnetic conductive gasket and tapered block. When the needle moves up and down in the nozzle, the needle can be regarded as a cylinder. While the cylinder moves in fluid, the viscous damping of fluid to the cylinder can be calculated as follows,


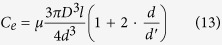


Where *C*_*e*_ is viscous damping of glue. *d* is the diameter of the needle (*d* = 6 mm), *μ* is the kinematic viscosity. *d*^*′*^ is the diameter of the glue channel of the nozzle.

Damping among the GMM rod, magnetic conductive gasket and tapered block can be calculated as follows,


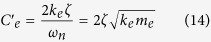


Where *k*_*e*_ is the total equivalent stiffness of the system, *m*_*e*_ is the total equivalent mass of the system, *ω*_*n*_ is the natural frequency, and ζ is the damping ratio (ζ = 0.25).

The equivalent damping can be expressed as follows,


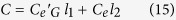


### Fluid-solid coupling model

Assume glue in the dispenser is power-law fluid. Glue in the nozzle chamber follows continuity equation, momentum conservation equation and constitutive equation. As the chamber formed between the needle and the nozzle is axial symmetry, the control equation in cylindrical coordinates of glue in the chamber is as follows,


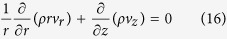



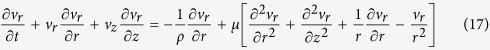



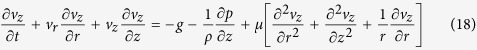



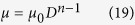


Where *v*_*r*_ is the radial component of glue flow velocity, *v*_*z*_ is the axial component of glue flow velocity, *p* is pressure in syringe, *μ*_*0*_ is nominal viscosity, *n* is power index of the power law fluid, *D* = *(I*_*2*_)^*1/2*^, *I*_*2*_ can be calculated as follows,


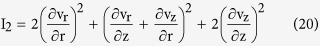


**Figure 5:**
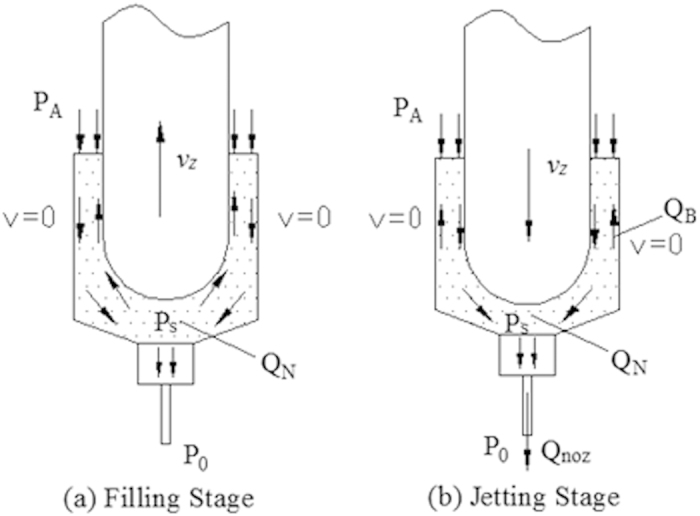
The glue’s flowing process.

Glue in the nozzle chamber flows when the needle moves. On the basis of the work principle, the glue’s flowing process can be divided into filling stage and jetting stage, as shown in Fig. 5. The proposed jetting dispenser is needle-type one, which is driven by mechanical collision. The fluid chamber is filled with liquid driven by air pressure, then the needle driven by driving force moves downwards and oppresses the fluid moving downwards. Meanwhile the high pressure is formed between the base and needle. The motion of the needle makes adhesives in the chamber shear-thinning. The collision between the needle and the base cuts off the glue and makes the high viscosity adhesives being jet from the nozzle while the partial high pressure reaches the maximum value.

**Figure 6:**
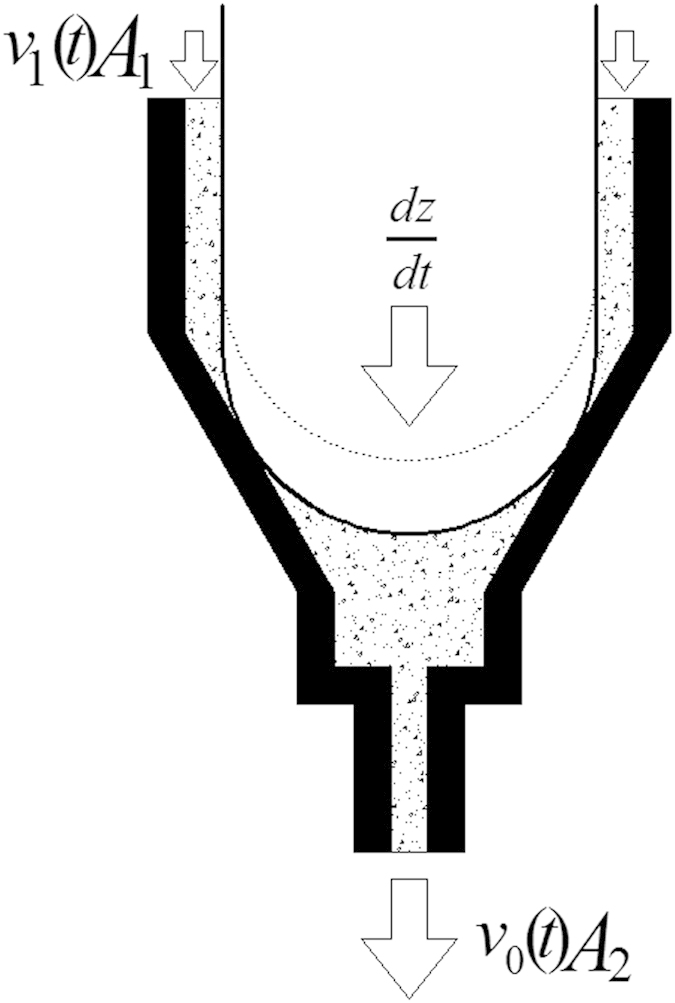
The analysis model of the jetting dispensing driven by mechanical collision.

In order to analyze the glue flow condition in a jetting period, the needle and fluid chamber are simplified as a two-dimensional axisymmetric mode as shown in Fig. 6.The diameter of the nozzle is *d*_*0*_, the diameter of needle head is *d*_*1*_, the diameter of fluid chamber is *D*, the displacement of needle movement is *Δl*.

During the needle movement, the fluid in chamber and nozzle satisfies the continuity equation and Navier-Stokes equation.


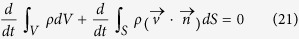



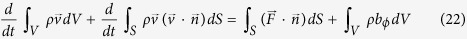


Where *V* is the fluid unit, *V* is the velocity of the flow, *ρ* is the density of fluid, *t* is the time, *F* is the force acting on plane S, *b*_*Φ*_ is the source term of scalar function.

The flow velocity of fluid in the chamber and nozzle is low and the effect caused by viscous resistance is little, so the continuity equation of no viscosity fluid movement could be described this process. The needle of the periodic reciprocating motion is simplified as a semicircle border, which diameter is *D* and velocity is *dz/dt*, the flow velocity in the nozzle and the entrance of fluid is *v*_*1*_*(t)* and *v*_*0*_*(t)* respectively. The flow pressure in fluid chamber is *P*, so the continuity equation could simplified as,


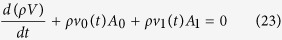


Where *A*_*0*_ is the cross-section area of nozzle and *A*_*1*_ is the cross-section of needle head, *V* is the volume of chamber before needle movement. The fluid flow in the hole of nozzle is differs from it in fluid chamber. The velocity of it is greater than the former, so the viscous resistance is the key factor affecting dispensing. The fluid flow in nozzle could be viewed as Hagen-Poiseuille flow in the thin pipe. The continuity equation could be satisfied automatically under this circumstance. The convection item of the momentum equation is zero, so the Navier-Stokes equation in *Z* axis could be simplified as,


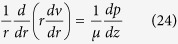


The boundary condition of nozzle wall is *V*_*r*_ = _*d/2*_ = 0, so the flow of jetting dispensing from nozzle could be concluded as,


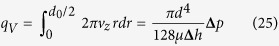


Where *ΔP* = *P* − *P*_*0*_, *P* is the internal pressure of nozzle chamber, *P*_*0*_ is the external pressure of nozzle chamber, which value is equal to atmospheric pressure. *d* is the diameter of droplet. The internal pressure *p* in nozzle chamber affects the dispensing velocity of fluid and the internal pressure *p* is determined by needle moving and extruding fluid chamber. So the jetting dispensing simulation could be researched according to changing the motion parameters of needle.

At jetting stage, the needle moves downward at speed *V*_*z*_. The needle motion makes the glue flow downward. The flow rate can be calculated as follows:


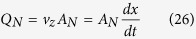


Where *Q*_*N*_ is the flow leaded by needle’s motion. *A*_*N*_ is the effective area of the needle, which would affect the pressure between the ball-shaped end of needle and the cone surface of nozzle (*P*_*s*_) directly. Then, *A*_*N*_ would affect the dot size finally. *Q*_*N*_ consists of backflow flow from the nozzle chamber (*Q*_*B*_) and dispensing flow rate from the nozzle hole (*Q*_*noz*_).When the needle moves downward, the motion makes *P*_*S*_ > *P*_*A*_. Then, the glue between the ball-shaped end of needle and the cone surface of nozzle would flow back. Because volume of the duct between the needle and the nozzle is small, glue in the duct is assumed to be incompressible and follows the following conservative law of the flow.


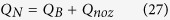


Flow velocity at the outlet of nozzle can be calculated as follows^20^,


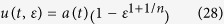


Where *u* (*t*, *ε*) is time-dependent flow velocity at the dimensionless radius *ε*, which is defined as *ε* = *r*/*R*_*noz*_. There is a corresponding *ε* to a location in the nozzle outlet. In laminar flow, when *ε*  = *0*, *u(t,0)* is the maximum velocity, *u*(*t*, *ε*) *=* *a(t). R*_*noz*_ is the radius of the nozzle outlet, which may affect dot size. *n* is the flow behavior index. *a(t)* is flow velocity at the center of the nozzle outlet, obtained from the following equation^21^,


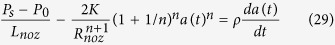


*P*_*0*_ is the atmospheric pressure. *L*_*noz*_ is the length of the nozzle outlet, *L*_*noz*_/*R*_*noz*_ may determine the viscosity range that the nozzle can adapt to. *K* is the consistency index. *ρ* is density of the glue.

The jetting flow rate at outlet of the nozzle can be expressed as follows^22^,


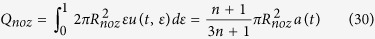


The pressure drop of the back flow is expressed as follows,


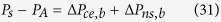


Where *P*_*A*_ is pressure in the duct between the needle and the nozzle, *ΔP*_*ce,b*_ is the pressure drop of the back flow due to flow contraction and expansion, *ΔP*_*ns,b*_ is the pressure drop of the back flow through the annular duct between the needle and the nozzle. *ΔP*_*ce,b*_ and *ΔP*_*ns,b*_ can be calculated as follows^22^,


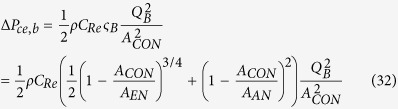



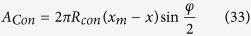



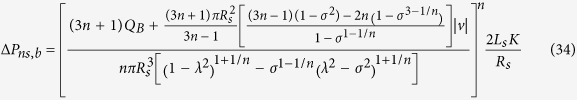



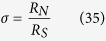


Where *ζ*_*B*_ is the local loss factor of the back flow due to contraction and expansion. *C*_*Re*_ is the empirical correction factor of the flow contraction and expansion loss coefficient, which depends on the Reynolds number of the flow at the contraction section. *L*_*s*_ and *R*_*s*_ are the length and radius of the duct between the needle and the nozzle. σ is the dimensionless radius of the needle. *R*_*N*_ is the radius of the needle. *λ* is the ultimate dimensionless radius of the back flow, which is a function of *n* and σ. *φ* is the cone angle of the nozzle. *A*_*con*_ is the area of the cone surface between the needle and the nozzle, which is determined by *R*_*N*_ and *φ. A*_*con*_ also may affect dot size. *A*_*AN*_ is the cross-sectional area of the duct between the needle and the nozzle.

When the needle moves downward, the force it bears from glue consists of damping force and upward pressure, which can be expressed as follows:


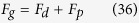


Where *F*_*g*_ is the force the needle bears from glue. *F*_*d*_ is damping force. *F*_*p*_ is upward pressure. *F*_*d*_ can be calculated as follows:


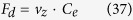


*F*_*p*_ can be calculated as follows,


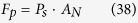


### Simulink model

**Figure 7:**
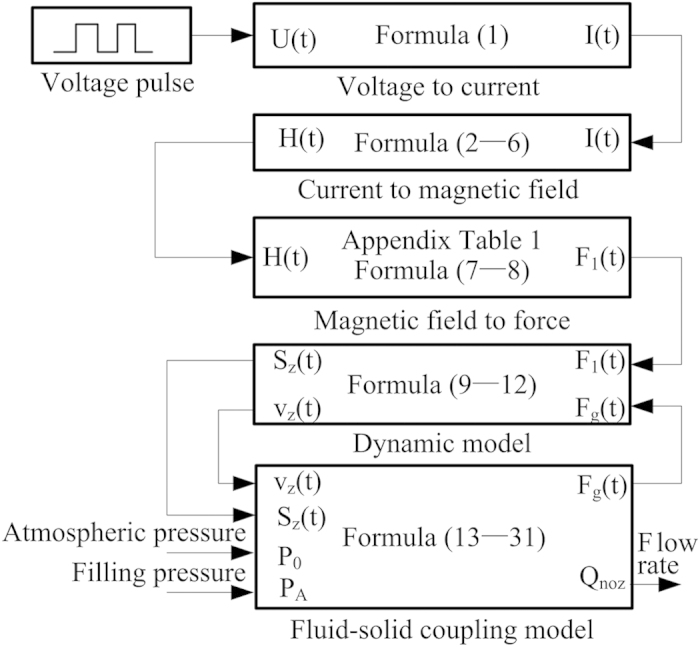
Simulation flow chart.

The numerical simulation is presented with Matlab-Simulink. The Simulink model consists of circuit model, electro-magneto-displacement model, dynamic model, and fluid-solid coupling model. Assume that glue in dispenser is incompressible and continuous, the dynamic characteristics of which follows the rheological power-law model. In the Simulink model, the boundary conditions contain voltage waveform used to activate the coil, atmosphere pressure and filling pressure in syringe. The amplitude of the current waveform is 6 A. The frequency of the waveform is 50 Hz. The duty circle of the waveform is 40%. The atmosphere pressure is 0.1 MPa. The filling pressure in the syringe is 0.25 MPa. The chart of the system model is shown in Fig. 7. The parameter list of the model is presented as shown in Table 2.

## Results

**Figure 8:**
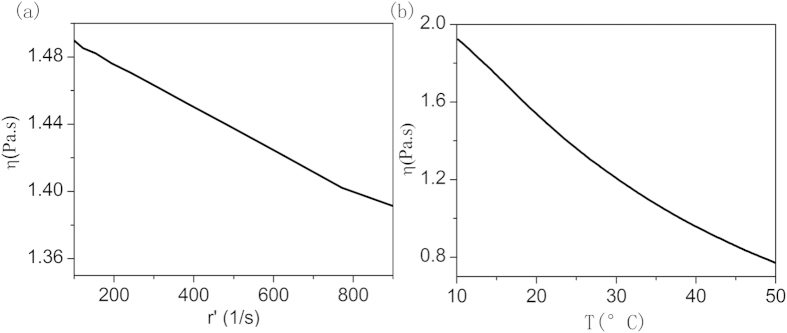
Rheological properties of the adhesives. (**a**) is the viscosity-shearing rate curve and (**b**) is viscosity-temperature curve.

The dispensing experiment is carried out under room-temperature, following the simulation. The commercial silica DC-OE-6336 is used in the dispensing experiment. The A and B components are mixed following proportion of 1:1. The rheological properties are tested by the rheometer, and the 60 mm diameter plate is used. The viscosity-shear rate curve is presented as Fig. 8(a), while the temperature is 25 degree Celsius, and the viscosity-temperature curve is presented in Fig. 8(b), while the shearing rate is 1000 s^−1^. It is obvious that the viscosity of the adhesive is not sensitive to the shearing; however it is very sensitive to the temperature. For this kind of adhesives, a preheating part is significant with the jet dispenser. A preheating chamber accompanying with jet dispenser will be designed and examined in the near future. On the contrary, the shearing sensitive adhesives could be easier jet by the dispenser on room temperature. There are also some other method to lower viscoelasticity of the adhesives for smooth dispensing, for example ultrasound^23^.

**Figure 9:**
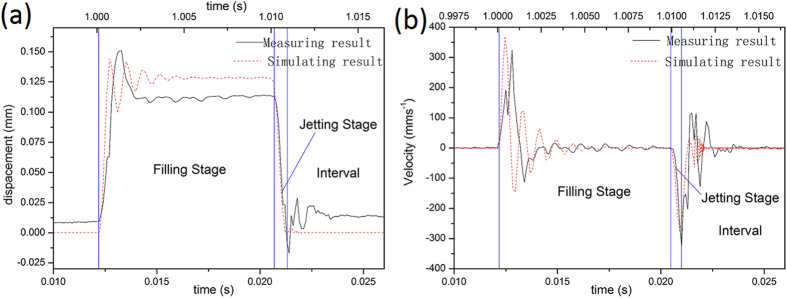
The displacement curves from (**a**) and the velocity curves from (**b**).

Figure 9(a) shows the displacement curve of the needle, and Fig. 9(b) shows the velocity curve of the needle. As shown in Fig. 9(a), the maximum displacement of the simulating result is 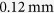, and the measuring result is 0.11 mm. Figure 9(b) indicates that when the needle moves upward, the maximum simulated velocity is 370 mm•s^−1^, and the maximum measured velocity is 330 mm•s^−1^; When the needle moves downward, the maximum simulated velocity is 290 mm•s^−1^, and the maximum measured velocity is 320 mm•s^−1^.

There is a close correspondence between the needle displacement and velocity curves computed in the simulation and those observed in the experiment. The correspondence proves the simulation models and the method are correct. When the needle moves up and keeps at the peak position, glue in the syringe flow into the nozzle chamber. This is filling stage of the dispenser work flow. After the filling stage, the needle moves downward rapidly. The needle motion makes adhesives flowing and the pressure between the ball-shaped end of needle and the cone surface of nozzle (*P*_*s*_) increasing. Then some adhesives are jet out and the dot is formed. After an interval the next circle follows.

**Figure 10:**
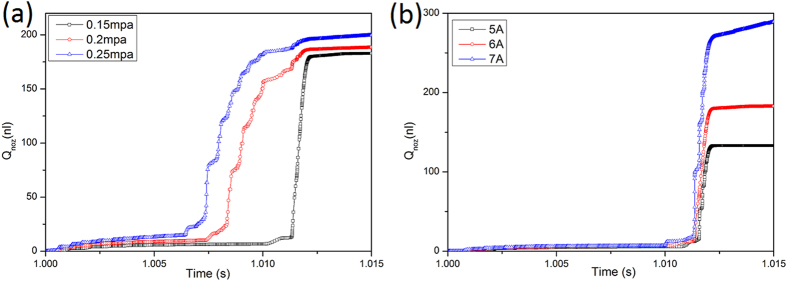
Q_noz_ curve of different P_A_ from (**a**) and Q_noz_ curve of different current from (**b**).

Figure 10(a) presents the alteration of dispensing flow rate within one circle, when the current in the coil is 6A and the filling pressure—*P*_*A*_ is 0.15 MPa, 0.2 MPa and 0.25 MPa respectively. As shown in the figure, the dispensing flow rate increases from 180 nl to 200 nl with *P*_*A*_ increasing. Figure 10(b) depicts the alteration of dispensing flow rate within one circle, when *P*_*A*_ is 0.15 MPa and the current in the coil is 5A, 6A and 7A respectively. The dispensing flow rate increases from 140 nl to 280 nl with the current increasing. From the two figures, it is obvious that effect of the current on the dot size is larger, compared with the filling pressure. Variation of the filling pressure and the current may lead to the change of the dot size. The appropriate dot size can be obtained by adjusting the current or the filling pressure. The power consumption of the dispenser will become larger with the current increasing. Filling pressure is preferred parameter to adjusting the dot size. If the flowing velocity at the center of the nozzle outlet can reach up to a critical value, the glue flow is expected to form dispensing dots. The critical value in other references is considered as 10 m/s. The flow rate of per circle at the outlet is nearly equal to the dot size. In dispensing experiment, the volume of 10 thousand dots is about 3 ml, when *P*_*A*_ is 0.25 MPa and the current is 6A. The volume of one dot is about 300 nl.

**Figure 11:**
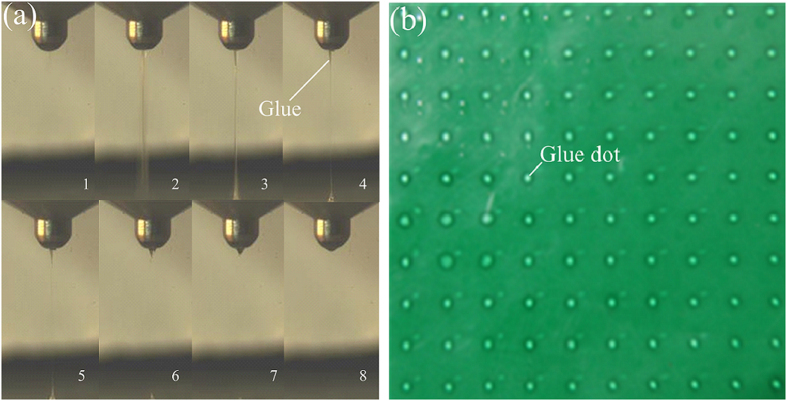
Dot forming process from (**a**) and jetting dot matrix from (**b**).

One cycle of dot forming process recorded with high speed camera is illustrated in Fig. 11(a). The first picture is the beginning of the jetting stage. Following, the second picture depicts glue is jet out and the neck appears. Then, the picture 3, 4 and 5 show the radius of the filament becoming smaller. The sixth picture shows the filament breaks off. The seventh picture depicts the droplet at the bottom forms the dot and recoil goes back to the nozzle. And the last picture presents the end of the jetting stage.

The 10×10 dots matrix jet by the dispenser is shown in Fig. 11(b). The highest work frequency of this kind of dispenser can reach up to 250 Hz. The duty circle of the control signal is 50%. The current in the coil is measured by oscilloscope. The current probe of the oscilloscope is set as 100 mV presenting 1 A. The current amplitude is 5.12 A.

The simulation and experiment results prove the proposed dispenser driven by GMM can work steadily, and also easily change the dot size. However, if viscosity of glue is greater than 1.5 Pa•s, the dispensing experiment shows the dispenser cannot work. The proposed dispenser is driven by GMM, so high frequency is a significant advantage of the novel dispenser. The stroke of the needle is limited by GMM, so it is difficult for the proposed dispenser to jet high viscosity adhesives at room temperature.

Contact-based dispenser can deliver over one hundred dots of glue per second^24^. Pneumatic jetting dispenser (DJ-2100) of Asymtek can operate 100 dots per second^25^. The work frequency of EFD Picodot serials dispenser driven with PZT can reach 65 Hz^22^. Compared with these commercial valves, the new GMM-based jetting dispenser significantly increases to 250 Hz.

**Figure 12:**
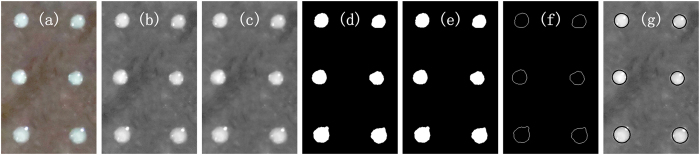
Image processing steps. (**a**) is original image, (**b**) is gray, (**c**) is Gaussian filtering, (**d**) is threshold segmentation, (**e**) is region filling, and (**f**) is contour lines extraction, (**g**) is least-squares circle fitting.

The diameter represents the volume of the fluid dot delivered by the new dispenser. Dot diameter is tested with the image processing method as shown in Fig. 12, where an image of 2 × 3 dots matrix is presented. Firstly, the original image is load to memory. Following image graying, noises are reduced by Gaussian filtering. Then threshold segmentation, contour lines extraction and region filling are operated. At last, the diameter can be gotten by least-squares circle fitting.

**Figure 13:**
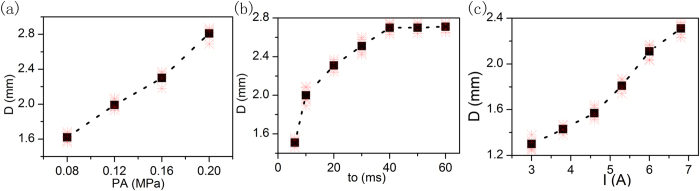
Dot size variation curve. (**a**) is effects of the filling pressure, (**b**) is effects of the opening time and (**c**) is effects of the current.

The effects of the filling pressure in the syringe to the dot size are as follows. While the activating current is 6.8 A, the valve opening time is 20 ms, and the filling pressure is adjusted to 0.08 MPa, 0.12 MPa, 0.16 MPa and 0.2 MPa respectively, the dot size is tested. The testing results are presented in Figure 13(a). It is obvious that the fluid dot size increases with the filling pressure becoming higher. With the increasing of filling pressure, there will be more fluid accumulated in the chamber of the nozzle, so the dot size becomes larger.

The effects of the valve opening time to the dot size are as follows. The valve opening time is controlled by the voltage waveform. While the filling pressure in the syringe is 0.15 MPa, the activating current is 6.8 A, and the opening time of the valve is set as 6 ms, 10 ms, 20 ms, 30 ms, 40 ms , 50 ms, and 60 ms respectively, the dot size is tested. The testing results are presented in Fig. 13(b). It is obvious that the fluid dot size increases with the opening time of valve becoming longer. With the opening time of the valve becoming longer, there will be more fluid accumulated in the chamber of the nozzle, so the dot size becomes larger. While the opening time of the valve exceeds 40 ms, the dot size will not increase, as the adhesives have been filled to the full of the nozzle chamber within 40 ms. Trends of the dot size is consistent with the simulation results.

The effects of the current to the dot size are as follows. While the s filling pressure in the syringe is 0.15 MPa, the valve opening time is 20 ms, and the current is adjusted to 3 A, 3.8 A, 4.6 A, 5.3 A, 6.0 A and 6.8 A respectively, the dot size is tested. The testing results are presented in Fig. 13(c). It is obvious that the fluid dot size increases with the current becoming larger. With the increasing of current, the stroke of the needle will increase, and the needle can get more kinetic energy in jetting stage, so the dot size becomes larger.

**Figure 14:**
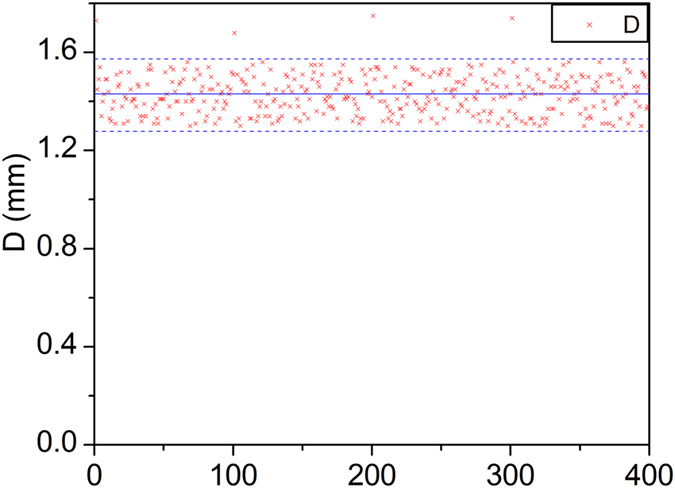
Diameter scatters of the adhesive dots.

Figure 14 shows the diameter scatters of the adhesive dots. The diameter represents the volume of the adhesive dot. The diameter is measured with the image processing method. Four 10 × 10 dots matrixes are measured. Most of the diameter scatters in 1.3−1.57 mm. However, there 4 dots (1st dot, 101st dot, 201st dot and 301st dot) diameter exceed 1.57 mm. Volume of the first dot jet by the dispenser after an interval is large. The accumulation of adhesives at the nozzle exit may lead to the problem. The issue still has been studied till now. If get rid of the 4 dots, the sample variance is 0.0062 mm^2^, and the diameter errors is within ±10%.

The reliability and the life-span of the jet dispenser have been assessed. The jet valve has worked over 50 million times till now, and it is still ok. The new jet valve reaches the level of the commercial jet valve.

## Conclusions

### 

The innovative jetting dispenser can deliver 250 dots of glue per second by using the high density GMM drive combining with the magnifying structure, and exceeds the jetting frequency of the other dispensers. System dynamic behaviors are evaluated by simulation and measurement. The maximum velocity of jetting needle reaches 330 mm•s^−1^ -tested, and the maximum displacement of jetting needle is 0.11 mm-tested. Moreover, the simulation model of the new system is established by using Matlab-Simulink combining with circuit model, electro-magneto-displacement model, dynamic model, and fluid-solid coupling model. It provides the theoretical analysis system for developing the new jetting dispenser. In a word, the GMM-based dispenser will provide principle & technical prototype for high-performance jetting.

## Additional Information

**How to cite this article**: Zhou, C. *et al.* The principle and physical models of novel jetting dispenser with giant magnetostrictive and a magnifier. *Sci. Rep.* **5**, 18294; doi: 10.1038/srep18294 (2015).

## Figures and Tables

**Table 1:** GMM strain data.

Magnetic intensity (kA·m^−1^)	Strain (ppm)
−2.660	−584.9
1.845	−631.5
7.477	−587.3
12.771	−386.9
17.286	−130.5
23.929	97.6
31.224	262.5
36.099	358.9
43.275	423.3
60.000	521.6
73.949	592.2
87.941	643.1
101.778	682.6
116.705	718.9
129.773	747.5
147.017	774.9
160.441	801.5
174.056	825.2
188.852	847.4
203.353	866.0

**Table 2:** Parameter list of the model.

Parameter	Symbol	Values	Unit
Eddy current constant	*τ*	0.0005	s
Length of GMM rod	*L*_*G*_	0.06	m
Cross-sectional area of GMM rod	*A*	7.85 × 10^−5^	mm^2^
Relative permeability of GMM	*μ*_*r*_	8.5	–
Inner cylindrical surface area of the coil	*S*_*in-coil*_	8.65 × 10^−5^	mm^2^
Stiffness of GMM rod	*kG*	1.07 × 10^8^	N·m^−1^
Young modulus of GMM	*E*_*y*_	1.5 × 10^10^	Pa
Mass of the lever	*m*_*f*_	50.6	g
Mass of the needle	*m*_*z*_	17.6	g
Mass of the GMM rod	*m*_*g*_	43.4	g
Stiffness of the spring	*k*_*t*_	20.6	N·mm^−1^
Flow behavior index	*n*	0.57	–
Length of the nozzle outlet	*L*_*noz*_	0.8	mm
Radius of the nozzle outlet	*R*_*noz*_	0.1	mm
Density of the glue	*ρ*	1780	kg·m^−3^
Diameter of the needle	*d*	6	mm
Filling pressure	*P*_*A*_	0.15	MPa
